# Heritability of DNA-damage-induced apoptosis and its relationship with age in lymphocytes from female twins

**DOI:** 10.1038/sj.bjc.6603257

**Published:** 2006-07-04

**Authors:** R S Camplejohn, S Hodgson, N Carter, B S Kato, T D Spector

**Affiliations:** 1 King's College London School of Medicine, Department of Oncology, Rayne Institute, St Thomas’ Hospital, London SE1 7EH, UK; 2 Division of Clinical Developmental Sciences, St Georges, University of London, Cranmer Terrace, London SW17 ORE, UK; 3 King's College London School of Medicine, Twin Research & Genetic Epidemiology Unit, Division of Genetics, St Thomas’ Hospital, London SE1 7EH, UK

**Keywords:** apoptosis, ageing, twins, DNA damage, lymphocytes

## Abstract

Apoptosis is a physiological form of cell death important in normal processes such as morphogenesis and the functioning of the immune system. In addition, defects in the apoptotic process play a major role in a number of important areas of disease, such as autoimmune diseases and cancer. DNA-damage-induced apoptosis plays a vital role in the maintenance of genomic stability by the removal of damaged cells. Previous studies of the apoptotic response (AR) to radiation-induced DNA damage of lymphoid cells from individuals carrying germline *TP53* mutations have demonstrated a defective AR compared with normal controls. We have also previously demonstrated that AR is reduced as individuals age. Results from the current study on 108 twins aged 18–80 years confirm these earlier findings that the AR of lymphoid cells to DNA damage is significantly reduced with increasing age. In addition this twin study shows, for the first time, that DNA-damage-induced AR has a strong degree of heritability of 81% (95% confidence interval 67–89%). The vital role of DNA-damage-induced apoptosis in maintaining genetic stability, its relationship with age and its strong heritability underline the importance of this area of biology and suggest areas for further study.

Apoptosis is an essential mechanism for eliminating cells with DNA damage and thereby maintaining genomic stability (Zhivotovsky and Kroemer, 2004). A considerable number of genes involved in the cellular response to DNA damage have been identified and perhaps the most studied of these is the tumour-suppresser gene *TP53*, which in its wild-type form, is able to induce apoptosis when damage to DNA is too great to be repaired. The importance of this gene is emphasised by the fact that the *TP53* gene is mutated in more than half of all cancers and inactivated in many more (Hollstein *et al*, 1994; Soussi and Lozano, 2005). We have previously shown that peripheral blood lymphocytes (PBL) from carriers of germline *TP53* mutations have a defective apoptotic response (AR) when subjected to gamma radiation (Camplejohn *et al*, 1995, 2000, 2001). The reduction in AR is such that there is essentially no overlap in the response distributions in *TP53* mutation carriers and normal controls (12 *vs* 46% mean response, *P*<0.001 in the two groups in Camplejohn *et al* (2000)). This reduction in AR exactly mirrors that seen in thymocytes from heterozygous *TP53* knockout mice (Clarke *et al*, 1993; Lowe *et al*, 1993).

We have demonstrated previously that DNA-damage-induced AR of PBL is reduced with increasing age at a rate of around 3–5% per decade of life in both normal controls and cancer patients (Camplejohn *et al*, 2003). However, a weakness in this earlier study was a relative paucity of normal individuals over 45 years of age. Studies in rodents support the age-related reduction in DNA-damage-induced apoptosis in several tissues. For example, Polyak *et al* (1997) observed a reduced AR of lymphocytes to 5 Gy radiation in mice as they aged and Suh *et al* (2002) found a reduced AR in rat liver cells exposed to the DNA damaging agent methyl methane-sulphonate. Apoptosis plays a major role in the immune functioning of lymphocytes but the literature is conflicting concerning the changes in constitutive apoptotic levels of immune cells with ageing. However, there is considerable evidence that senescent, apoptosis-resistant lymphocytes may accumulate *in vivo*, as individuals age (Hsu *et al*, 2005).

In the present study, we wished to test whether the DNA-damage-induced AR had a significant genetic basis and to confirm in normal individuals with a wide range of ages that AR is indeed related to age. To do this we measured AR in 54 pairs of twins (28 monozygotic and 26 dizygotic) from the TwinsUK adult twin registry in London consisting of unselected twin volunteers developed to study the genetics of age-related disease (www.twinsUK.ac.uk).

## MATERIALS AND METHODS

### Subjects

Participants were female twins (28 monozygotic and 26 dizygotic pairs) from the TwinsUK adult twin registry, which consists of volunteers unselected for any disease (www.twinsuk.ac.uk). These twins have been shown to be similar to age-matched singletons in the population in terms of disease-related and lifestyle characteristics (Andrew *et al*, 2001).

### Apoptotic assay

**Figure 1:**
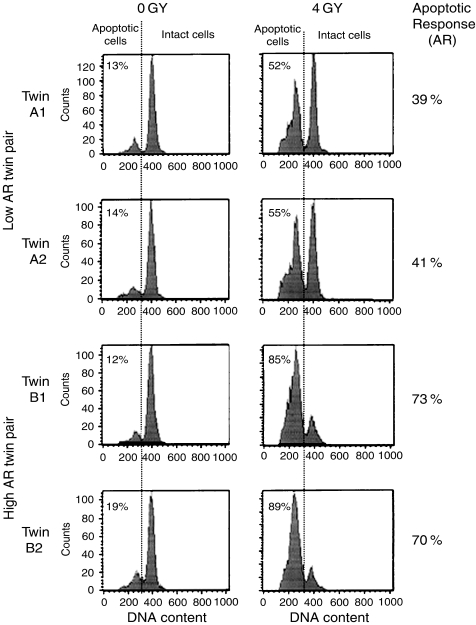
Apoptotic data for two identical twin pairs is shown above. The histograms were produced by gating data from 10 000 cells per plot using forward and 90° light scatter to remove contaminating monocytes and pulse height/width of the DNA signal to remove cell clumps from the analysis. The left hand panels illustrate results for mock-irradiated cells and the right-hand panels show data following 4 Gy irradiation. The AR for each individual was calculated by subtracting the percentage of apoptotic cells for mock-irradiated cells from the percentage following 4 Gy irradiation. As can be seen, twin pair A had an AR of around 40%, while twin pair B had an AR of 70%.

Peripheral blood lymphocytes were separated from 10 ml of blood by centrifugation on Histopaque and 5 × 10^6^ cells diluted in 10 ml RPMI culture medium with 10% serum was added to each T25 flask (Becton Dickinson, Oxford, England) and the cells were cultured for 70 h and either irradiated or mock-treated. Irradiation was carried out using a Gammacell 1000 Elite (Nordion International Inc., Ontario Canada) containing a caesium 137 source and with a dose rate of 858 cGy min^−1^. Cells were subjected to a dose of 4 Gy. Following a further 24 h culture, cells were split into two aliquots and fixed in 70% ethanol. After removal of ethanol, 2 × 10^6^ cell aliquots were subjected to treatment with 0.1 M HCl at 37°C for 12 min to extract low molecular weight DNA. After washes, cells were stained for DNA content by addition of propidium iodide (PI – final concentration 50 *μ*g ml^−1^) in a volume of 1 ml. Cells were stained for a minimum of 30 min before measurement of red fluorescence (PI), forward and 90° light scatter on a FACSCalibur flow cytometer (Becton Dickinson, Oxford, England). At least 10 000 cells per sample were scanned and data stored in list mode prior to analysis using CellQuest software. Doublet discrimination using pulse area/width analysis on the PI signal was used to remove cell clumps from the analysis. Measurement of the extent of apoptosis was performed by assessment of cells appearing in a sub-G1 peak on DNA profiles. The AR to radiation was defined as the increase in apoptosis seen when comparing the irradiated with the unirradiated sample (% apoptosis after 4 Gy – % apoptosis after 0 Gy). Examples of the apoptotic data for a high and low AR twin pair are illustrated in Figure 1. This flow cytometric method has been validated in many publications and by comparison in our laboratory with a number of other techniques including light and electron microscopic counting of apoptotic cells and flow cytometric cell sorting of apoptotic cells (Camplejohn *et al*, 1995, 2000). In studies of over 1200 individuals the method has been found to be reliable and reproducible.

### Modelling and statistical analysis

Apoptotic response was shown to be normally distributed and standard regression techniques were used to assess associations between age and AR, utilising the regression cluster option in StatCorp. (2003) to account for correlation within twin pairs. Apoptotic response heritability was modelled using structural equation modelling implemented using MX software applied to MZ and DZ twins (Neale and Maes, 1991). The best fitting model (see Table 1) included additive polygenic effects (*A*=81; 95% confidence interval is 67–89%) and unique environmental effect specific to the individual (*E*=19%; confidence interval is 11–33%). As it has been suggested that there may be a spreading of data with age (i.e., the difference between the two twins in a twin pair may increase with age), this was also assessed. The difference between AR for each twin pair was computed and this variable was called apoptotic difference. Subsequently, apoptotic difference was regressed on age.

## RESULTS

Figure 1 illustrates apoptotic data for two pairs of MZ twins, a low AR pair (age 65 years) and a high AR pair (age 18 years). The twins within each pair are in good agreement with each other and we found that there is no significant association between difference in AR and age (*P*-value=0.648). Thus, there is no evidence that with ageing there is a ‘spreading’ of results which would tend to make twin pairs discordant with age.

**Figure 2:**
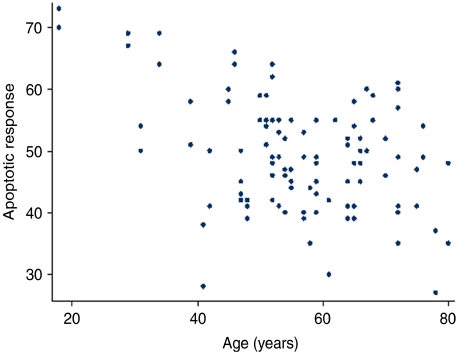
Relationship between AR to 4 Gy irradiation and age in years (a regression coefficient of −0.277, *P*-value=0.007, *R*^2^=0.1505).

The mean age of the twins was 57 years (range 18–78) for the monozygotic pairs and 56 years (range 29–80) for the dizygotic pairs. The mean ARs in the two zygosity groups were also very close, namely 50% (range 27–73) for the MZ twins and 49% (range 30–69) for the DZ pairs. Given these findings the two zygosity groups were analysed together in terms of the relationship between age and AR. As illustrated in Figure 2, there was a clear negative correlation between AR and age (*P*=0.007), with a reduction in AR of 3% per decade of life, which is similar to that found in earlier studies (Camplejohn *et al*, 2003).

**Figure 3:**
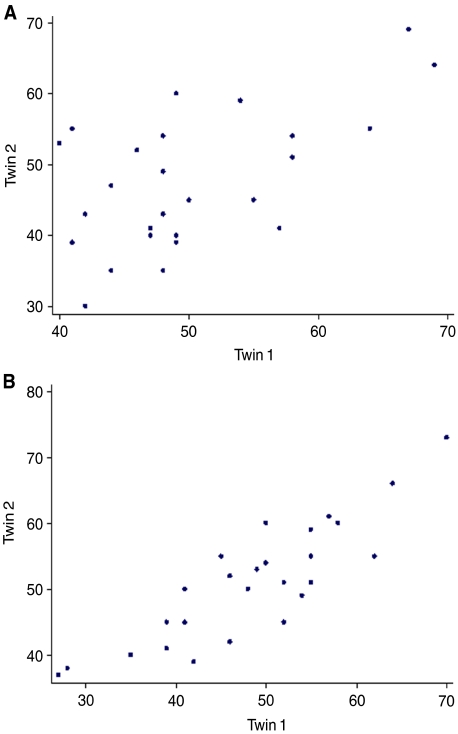
Illustrates the relationship between twin pairs for (**A**) DZ twins –correlation 0.58 *P*=0.002) and (**B**) MZ twins – correlation 0.87 (*P*=0.001).

When comparing the similarity between twin pairs, the higher intrapair correlations in MZ *vs* DZ pairs (see Figures 3A and B, respectively) is consistent with a clear genetic influence on AR. It was found that the additive genetic and environmental (AE) model, dropping common environment (C), fitted the data best and suggests a high heritability of 0.81 (95% confidence limits 67–89%) with the remaining 19% being attributed to specific environmental effects or random error (see Table 1). Clearly, it is impossible to exclude some degree of influence on the data of common/shared environment of up to 10% and much larger data sets would be needed to exclude such an influence.

## DISCUSSION

In the current study AR had a high degree of heritability of above 80% and AR reduced significantly with increasing age. These findings provide interesting insights into fundamental aspects of cell biology. DNA-damage-induced apoptosis is a primary mechanism to protect the organism from general genetic instability and the consequences thereof such as the development of malignant disease. *TP53* is the gene most often mutated in human cancer, and several studies have shown that loss of one allele in the germ line leads not only to a much increased risk of cancer but also to a decreased AR in lymphoid cells from both rodents (Clarke *et al*, 1993; Lowe *et al*, 1993) and humans (Camplejohn *et al*, 1995, 2000). In addition, a variety of other apoptosis related genes are mutated, or in other ways dysfunctional in many cancers. Such genes include Bcl2 but also include a range of both proapoptotic genes (downregulated) and antiapoptotic genes (upregulated) (Ding and Fisher, 2002; Hersey and Zhang, 2003).

The fact that AR appears highly heritable in humans is consistent with a body of research in rodents that demonstrates significant strain differences in DNA-damage-induced apoptosis in various tissues (for a review see Coates *et al*, 2005). The reduced AR seen in some mouse strains such as DBA/2 has been correlated with an increased incidence of genetic defects in irradiated cells (Watson *et al*, 1997; Wallace *et al*, 2001). The demonstration of a strong level of heritability for AR in lymphocytes from human volunteers in our study, along with the confirmation of an age-related decrease in AR, are important findings that suggest a number of further avenues of research. For example, does a low age-adjusted AR correlate with an increased disease risk and is there any way in which the age-related reduction in AR can be linked aetiologically with the increased risk of cancer and other diseases seen with increasing age? Does the level of age-adjusted AR correlate with lifestyle and other risk factors such as smoking or obesity. Such correlations with lifestyle factors have been demonstrated for telomere length another parameter, which is related both to the maintenance of genetic stability and age (Valdes *et al*, 2005)? In the current study telomere length was available for the individuals from which we measured AR and though the two parameters are both inversely related to age, there appeared to be no correlation between telomere length (measured in duplicate by Southern Blot with a CV<1%) and AR (data not shown – correlation coefficient=0.006, *P*=0.45). This lack of correlation between telomere length and AR suggests that AR may not be associated with the same risk factors as TRF but with other, as yet unknown, lifestyle and disease-related factors. It would also be consistent with the idea that AR is controlled by different genes and processes to those that control telomere length.

Apoptosis plays a central role in the maintenance of genetic stability and apoptotic defects are involved in various common diseases. Given these facts and the demonstration that we can measure the phenotype accurately in populations, a large scale family based or twin study is now needed to unravel the strong underlying genetic basis of the AR to DNA damage and to investigate environmental influences on this process.

## Figures and Tables

**Table 1:** Apoptotic response

**Model**	**A**	**C**	**D**	**E**	**df**	**−2LL**	**Δ*X*^2^**	**Δdf**	***P*-value**
ACE	0.5118	0.3007	—	0.1875	102	741.994			
ADE	0.8137	—	0	0.1863	102	742.998			
**AE**	**0.8138**	**—**	**—**	**0.1862**	**103**	**742.998**	**1.004**	**1**	**0.316**
CE	—	0.6958	—	0.3042	103	747.027	5.033	1	0.025

Apoptotic response (AR) heritability model estimates; A=additive polygenic effects; C=familial common environment; D=dominance effects; E=specific individual effects and measurement error. Model fit statistics for models AE and CE are presented as submodels of ACE. The best fit model is highlighted in bold.
